# A causality between fruit consumption and colorectal cancer: a two-sample Mendelian randomization analysis

**DOI:** 10.3389/fonc.2024.1362269

**Published:** 2024-03-01

**Authors:** Li Yang

**Affiliations:** Department of Gastroenterology, Shapingba Hospital Affiliated to Chongqing University, Chongqing, China

**Keywords:** colorectal cancer, fruit consumption, Mendelian randomization, two-sample MR, single nucleotide polymorphisms

## Abstract

**Background:**

Colorectal cancer (CRC) significantly threatens human health with increasing incidence and mortality. A debate continues whether fruit consumption is associated with CRC, despite dietary habits having an impact on the disease. The study aims to examine the causal relationship between fruit consumption and CRC based on a two-sample Mendelian randomization method (MR).

**Methods:**

Summary statistics for fruit consumption and CRC were obtained from the UK Biobank and the FinnGen Consortium, respectively. Analysis methods used in this study included the inverse-variance weighted (IVW), MR Egger, weighted median, simple mode, and weighted mode. Heterogeneity and horizontal pleiotropy were also assessed. Additionally, a leave-one-out analysis was performed to validate the robustness of the results.

**Results:**

We found that fruit consumption was associated with a reduction in CRC risk by the IVW method (*P* = 0.021). This protective effect was predominantly observed in males (OR 0.374; 95% CI: 0.157-0.892; *P* = 0.027), while no protective effect was noted in females. However, causal correlations were not observed upon analyzing 16 individual types of fruits. Moreover, our results were unlikely to be influenced by horizontal pleiotropy and heterogeneity. Leave-one-out analysis confirmed the stability of the results.

**Conclusion:**

Our findings suggest that a genetic predisposition for fruit consumption may be protective against CRC, underscoring the need for further research to elucidate the underlying mechanisms and dietary patterns involved.

## 1 Introduction

In 2020, there were an estimated 1.9 million new colorectal cancer (CRC) cases globally, accounting for 10.0% of all new malignant tumor cases, only after breast and lung cancer (1). Approximately 916,000 people died from CRC, representing 9.4% of all malignant tumor-related deaths, second only to lung cancer (2). Additionally, most CRC patients are diagnosed at an advanced stage due to no early symptoms or subtle signs (3). Moreover, the 5-year survival rate for most stage IV CRC patients is below 10% (4). CRC poses a significant threat to human life and health, emphasizing the importance of early prevention. Based on migrant epidemiology and etiology research, CRC incidence differs between Eastern and Western populations primarily due to dietary and nutritional factors (5). A healthy diet can prevent about 30-50% of CRC cases (6). Fruits are rich in dietary fiber, vitamins, and other potential anti-tumor bioactive compounds, making them possible protective factors against CRC (7).

Inconsistent results have been found in previous studies on the correlation between fruit consumption and CRC (8–13). Some studies linked higher fruit and vegetable consumption with lower CRC mortality rates. A meta-analysis indicated a reduced risk of CRC associated with a high intake of citrus fruits, apples, watermelon, and kiwi (14). However, Aoyama et al. did not find a strong correlation between low or consistently low intake of vegetables and fruits and the risk of CRC (9). These conflicting research findings may be attributed to methodological differences, confounding factors, and variations in dietary habits. To mitigate potential confounding factors and enhance the robustness of empirical evidence, Mendelian randomization (MR) has been employed as a methodological approach. MR utilizes genetic variability as instrumental variables (IVs) for evaluating the causal association between an exposure and its corresponding outcomes (15, 16).

MR analysis assumes that genetic variations are randomly distributed in the population, similar to a randomized controlled trial. This method mitigates reverse causation and confounding biases in observational studies, providing a more precise representation of the exposure-outcome correlation (17). Moreover, many genetic variations are associated with the consumption of proteins, fats, and carbohydrates. These genetic variations not only affect the intake of individual nutrients but also the overall dietary preferences and patterns (18). As a result, this study investigates the risk of CRC associated with fruit consumption using MR. Genetic data were sourced from the Medical Research Council Integrative Epidemiology Unit (MRC-IEU) project.

## 2 Materials and methods

### 2.1 Study design and data resource

**Figure 1:**
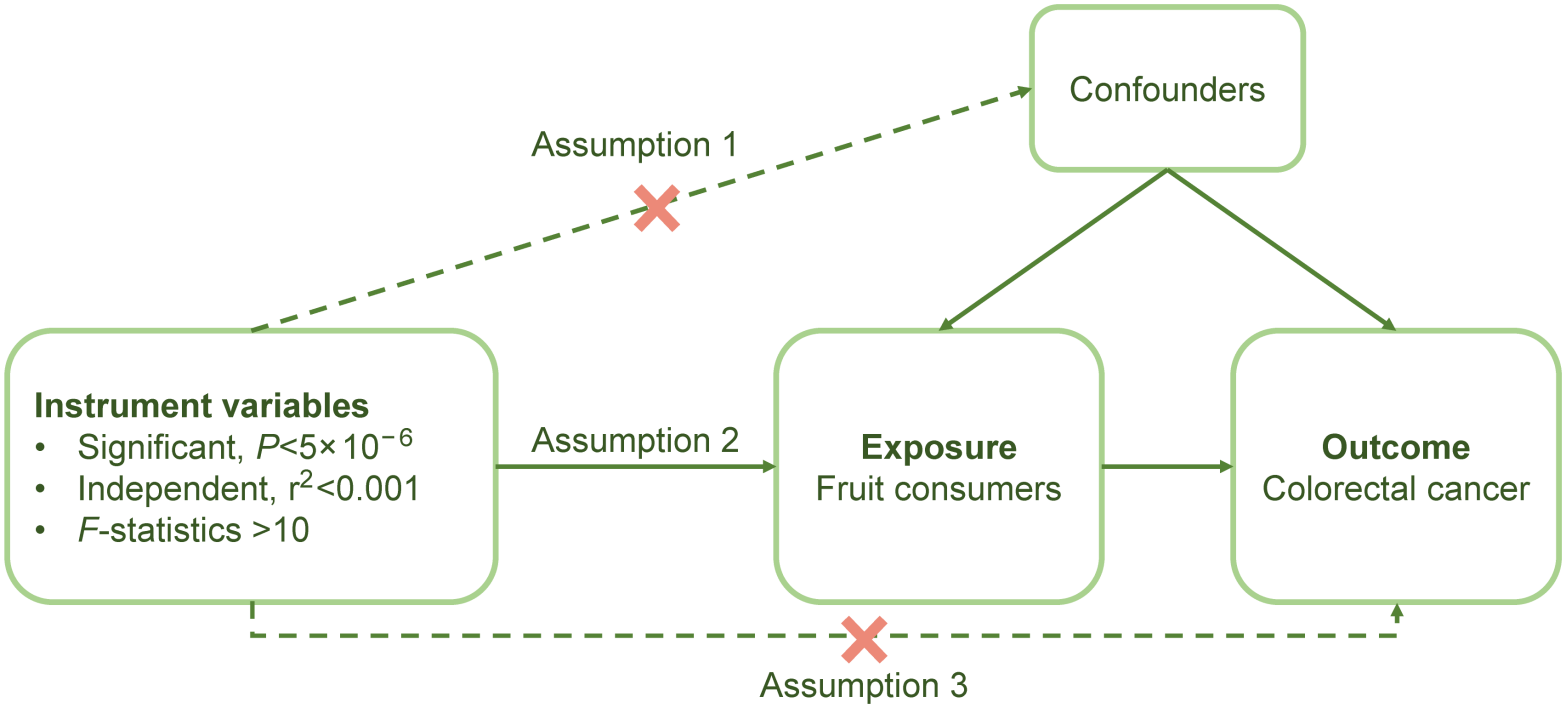
The process of MR analysis.

In this study, fruit consumption is considered the exposure variable, while CRC is regarded as the outcome. Single nucleotide polymorphisms (SNPs) have been selected as IVs for further analysis. The study adheres to the three fundamental assumptions of the two-sample MR design:

1. Assumption I: there is a significant association between genetic variants and exposure (*P* < 5 ×10^-6^, F-statistic > 10).
2. Assumption II: genetic variations are unrelated to any confounding factors within the exposure-outcome correlation.
3. Assumption III: genetic variants solely impact the outcome by virtue of their connection to the exposure (**Figure 1**).

This MR investigation is based on publicly available GWAS datasets, thus obviating the need for additional ethical approval from institutional review boards.

The CRC GWAS summary data were acquired from the FinnGen consortium, comprising 3,022 cases and 174,006 controls, all of European ancestry who provided informed consent. In addition, summary data for fruit intake (females and males) was obtained from the IEU OPEN GWAS project (https://gwas.mrcieu.ac.uk/) with 64,949 individuals of European descent (including 11,960 controls) and encompassed 9,851,867 SNPs.

We obtained GWAS summary statistics for different types of fruit from GWAS meta-analysis, including: apple intake, banana intake, berry intake, cherry intake, grape intake, grapefruit intake, mango intake, melon intake, orange intake, peach/nectarine intake, pear intake, pineapple intake, plum intake, prune intake, satsuma intake, dried fruit intake.

### 2.2 Selection of instrumental variables

In order to perform MR analysis, it is imperative to adhere to three essential assumptions: relevance, independence, and exclusion restriction. Consequently, a rigorous selection process was applied to all IVs used for subsequent analysis. Initially, we identified SNPs closely linked to the exposure (*P* < 5 × 10^-6^) and excluded those with an F-statistic < 10, ensuring their significance and reducing the potential for weak IV bias.

The F-statistic utilized in this study is defined by the formula R^2^ × (N − K − 1)/(1 −R^2^) where R^2^ represents the variance in exposure explained by each IV. R^2^ = 2 × β^2^ × EAF × (1 − EAF)/(2 × β^2^ × EAF × (1 − EAF) + 2 × SE^2^ × N × EAF × (1 − EAF)), where β denotes the allele effect size, EAF signifies the effect allele frequency, K is the number of IVs, and N is the sample size of the GWAS study. Subsequently, to ensure the independence of IVs, SNPs with strong linkage disequilibrium were excluded (r^2^< 0.001, kb = 10,000). Thirdly, the chosen IVs were examined for associations with other phenotypes (http://www.phenoscanner.medschl.cam.ac.uk/) to further mitigate the potential impact of horizontal pleiotropy on the study. Finally, the datasets related to the exposure and outcome were harmonized to ensure that the effect alleles were consistently aligned.

### 2.3 Statistical analysis

In this study, all statistical analyses were conducted using R software (version 4.3.1) with the TwoSampleMR package. We assessed the causal correlation between fruit consumption and CRC using various methods, including the inverse-variance weighted (IVW) method (the primary approach), as well as MR Egger, weighted median, simple mode, and weighted mode methods. Significance for a causal effect between the exposure and outcome was defined as *P* < 0.05. The results from the MR analyses were reported as odds ratios (ORs) along with their corresponding 95% confidence intervals (CIs). To assess individual SNP heterogeneity, Cochrane’s Q statistic was employed. Heterogeneity was considered absent for *P* values exceeding 0.05. Additionally, we performed sensitivity analyses to identify possible horizontal pleiotropy and verify the consistency of associations. These analyses encompassed the weighted median method and tests for heterogeneity. The weighted median method yielded a consistent overall effect estimate when more than 50% of the IVs were effective. Furthermore, a leave-one-out test was conducted to confirm that the observed causality remained unaffected by individual SNPs.

Given that gender difference, we performed a gender-stratified MR analysis, aiming to avoid potential sexual bias. To minimize the potential interactions between different types of fruit and isolate the independent effect of each category, we conducted an MR analysis for each type of fruit and CRC.

## 3 Results

### 3.1 Instrumental variable selection

After the initial selection process, which involved identifying SNPs with strong relevance (*P* < 5 × 10^-6^, F-statistic > 10) and eliminating those exhibiting linkage disequilibrium (r^2^ < 0.001, kb = 10,000), we identified a set of 13 SNPs as preliminary IVs. The lowest F-statistic was 20.908, and a comprehensive overview of the F-statistics can be found in **Table 1**. It’s worth noting that despite conducting exclusion of SNPs associated with the outcome and potential confounding factors using Phenoscanner, no SNPs were excluded at this stage. Subsequently, after aligning the exposure and outcome data, we proceeded with these 13 SNPs as IVs for further analysis.

**Table 1:** SNPs used as instrumental variables from fruit consumers and colorectal cancer GWASs.

SNPs	Exposure					Outcome					Chr	Pos	F	R^2^
	Beta	Se	*P*-value	Effect allele	Other allele	Beta	Se	*P*-value	Effect allele	Other allele				
rs11217077	-0.012	0.002	3.00E-06	C	T	0.065	0.031	0.035	C	T	11	118746223	21.813	0.000335745
rs118052164	-0.040	0.009	2.80E-06	A	T	0.119	0.096	0.218	A	T	9	6978352	21.971	0.000338171
rs17818946	0.017	0.004	1.70E-06	G	A	-0.016	0.049	0.751	G	A	18	72390746	22.913	0.000352667
rs36013074	0.027	0.006	4.00E-06	A	G	0.092	0.097	0.339	A	G	6	151026080	21.264	0.000327293
rs4648553	0.012	0.002	5.50E-07	A	G	-0.015	0.029	0.612	A	G	1	3648879	25.091	0.000386174
rs4663869	-0.016	0.004	4.80E-06	G	A	-0.002	0.035	0.946	G	A	2	239175023	20.908	0.000321817
rs4836713	0.019	0.004	1.40E-06	G	A	-0.010	0.079	0.903	G	A	9	120998947	23.324	0.000359001
rs4923532	0.011	0.002	7.60E-07	G	A	-0.046	0.028	0.092	G	A	11	28401900	24.462	0.000376504
rs55809544	-0.014	0.003	4.40E-06	G	A	0.094	0.045	0.034	G	A	13	99119023	21.065	0.000324235
rs72867233	-0.048	0.010	8.20E-07	G	A	0.132	0.227	0.561	G	A	2	32616643	24.303	0.000374061
rs75417443	-0.016	0.004	3.50E-06	A	G	-0.018	0.059	0.757	A	G	12	20027525	21.501	0.000330947
rs76331651	0.024	0.005	1.80E-06	G	A	0.091	0.070	0.193	G	A	15	88441033	22.792	0.00035081
rs7937987	-0.012	0.003	3.40E-06	T	C	0.069	0.037	0.061	T	C	11	94690088	21.597	0.000332427

Se, standard error; SNP, single nucleotide polymorphism; Chr, chromosome; Pos, position.

### 3.2 Mendelian randomization analysis

**Figure 2:**
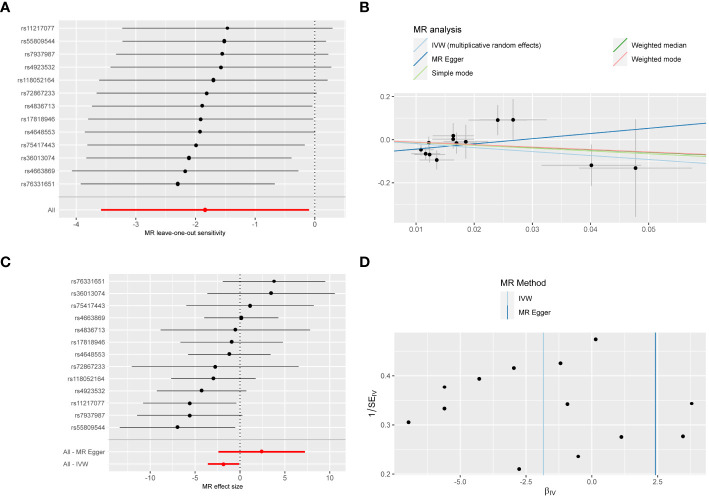
Sensitivity analysis **(A)**, scatter plot **(B)**, forest plot **(C)**, and funnel plot **(D)** of the causal effect of fruit consumption on colorectal cancer risk.

The genetic correlation between fruit intake and CRC was investigated using a random effects IVW approach. **Table 2** presents MR results and the five methods used in our analysis. Our results indicate that fruit intake is negatively correlated with CRC (OR 0.159; 95% CI: 0.033-0.759; *P* = 0.021) (**Figure 2**). According to the Cochrane’s Q test, there was no evidence of heterogeneity between fruit intake and CRC (*P* = 0.247). The MR-Egger method detects horizontal pleiotropy through its intercept with the y-axis. The presence of horizontal pleiotropy is indicated when the intercept is non-zero. To satisfy the exclusion restriction assumption, horizontal pleiotropy must not be present. No horizontal pleiotropy was observed in our MR analysis results (*P* = 0.094) (**Table 2**). Our MR analysis results were not influenced by individual SNPs (**Figure 2A**) based on the leave-one-out test.

**Table 2:** MR results of causal links between fruit consumers and colorectal cancer risk.

Exposure	Outcome	Nsnp	Method	β	Se	*P*-value	OR (95%CI)	Heterogeneity		Horizontal pleiotropy		
								Cochran’s Q	*P*-value	Egger intercept	Se	*P*-value
Fruit consumers	Colorectal cancer	13	MR Egger	2.414	2.459	0.347	11.183 (0.090-1386.369)	14.907	0.247	-0.068	0.037	0.094
			Weighted median	-1.170	1.145	0.307	0.310 (0.033-2.925)					
			IVW	-1.839	0.798	0.021	0.159 (0.033-0.759)					
			Simple mode	-1.309	2.145	0.553	0.270 (0.004-18.097)					
			Weighted mode	-1.139	1.795	0.538	0.320 (0.009-10.810)					

MR, Mendelian randomization; Se, standard error; OR, odds ratio; CI, confidence intervals; IVW, inverse variance weighted.

Scatter plots demonstrated that the causal association estimates from IVW, MR-Egger, and weighted median methods were similar, as judged by the slope of the line (**Figure 2B**). A forest plot (**Figure 2C**) visualizes the causal correlation between each SNP and CRC. The funnel plot, representing each SNP as an IV, showed that the derived causal effects were symmetrically distributed, suggesting a low probability of potential bias and indicating that the results were stable and reliable (**Figure 2D**). Therefore, these findings were stable and robust.

### 3.3 Gender-stratified MR analysis

To further explore the causal-effect difference of genders on fruit intake, a gender-stratified MR analysis was performed. In total, 15 SNPs were identified as IVs for gender-stratified fruit consumers, among which 8 SNPs were identified for fruit consumers in females, and 7 SNPs for males. The F statistics of IVs ranged between 21.054 and 28.781, indicating no evidence of weak instrument bias. Detailed information on these IVs is listed in **Supplementary Tables 3**, **4**.

**Figure 3:**
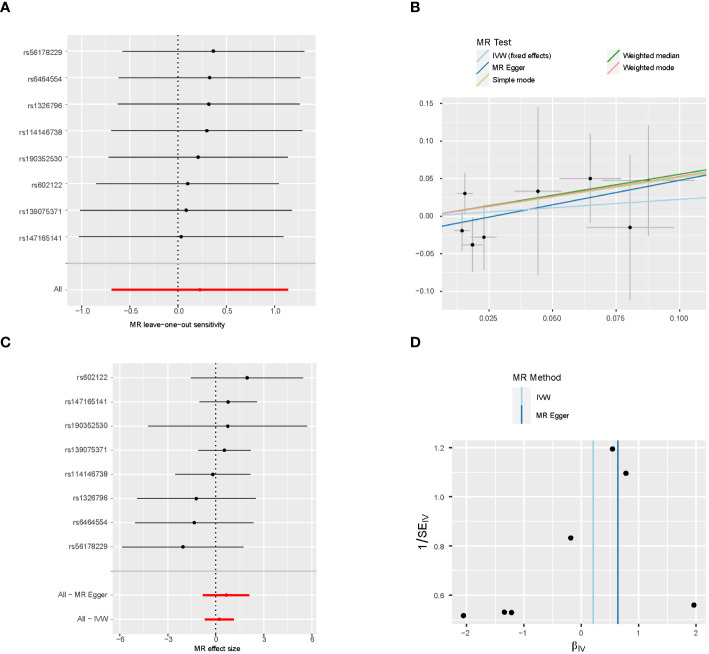
Sensitivity analysis of female **(A)**, scatter plot **(B)**, forest plot **(C)**, and funnel plot **(D)** of the causal effect of fruit consumption on colorectal cancer risk.

**Figure 4:**
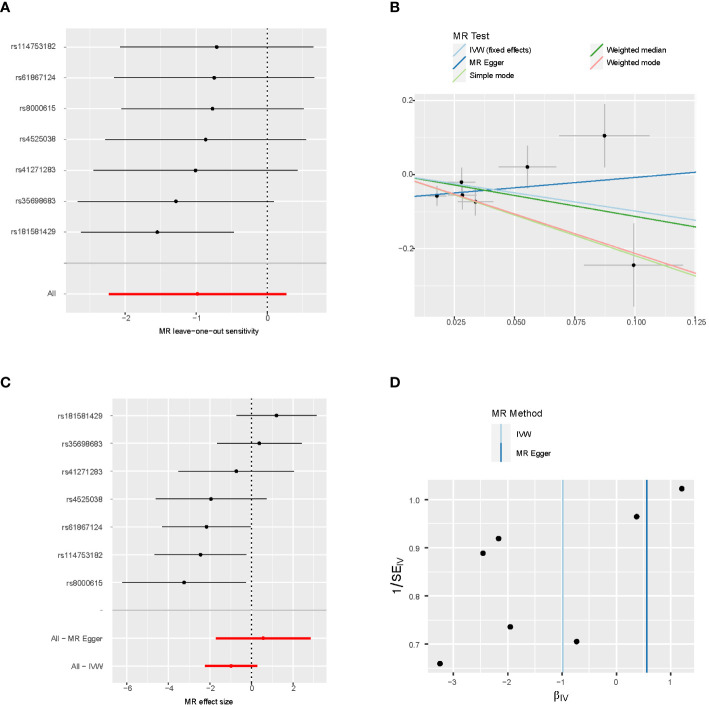
Sensitivity analysis of male **(A)**, scatter plot **(B)**, forest plot **(C)**, and funnel plot **(D)** of the causal effect of fruit consumption on colorectal cancer risk.

The MR results from gender-stratified fruit intake are listed in **Tables 3**, **4**. Gender-stratified MR showed that genetically predicted fruit intake was associated with CRC in males (OR 0.374; 95% CI: 0.157-0.892; *P* = 0.027). The scatter plots of IVs are shown in **Figures 3**, **4**. Moreover, the results from the Cochran’s Q and MR-Egger intercept tests are also shown in **Tables 3**, **4**. There is no heterogeneity was observed between the genetic IVs for fruit intake in both females and males, for which the FE-IVW method was used. MR-Egger intercepts did not detect any pleiotropy, indicating no evidence of potential horizontal pleiotropy (both intercepts *P* > 0.05). The leave-one-out analysis showed that the significant results were not driven by any single SNP (**Figures 3**, **4**).

**Table 3:** MR analysis of the impact of fruit consumption on CRC in females.

Exposure	Outcome	Nsnp	Method	β	Se	*P*-value	OR (95%CI)	Heterogeneity		Horizontal pleiotropy		
								Cochran’s Q	*P*-value	Egger intercept	Se	*P*-value
Fruit consumers in females	Colorectal cancer	8	MR Egger	0.655	0.736	0.408	1.925 (0.455-8.146)	4.271	0.748	-0.017	0.023	0.480
			Weighted median	0.559	0.594	0.346	1.750 (0.546-5.602)					
			IVW	0.225	0.465	0.628	1.253 (0.503-3.120)					
			Simple mode	0.515	0.866	0.571	1.674 (0.306-9.143)					
			Weighted mode	0.532	0.607	0.409	1.703 (0.518-5.594)					

MR, Mendelian randomization; Se, standard error; OR, odds ratio; CI, confidence intervals; IVW, inverse variance weighted.

**Table 4:** MR analysis of the impact of fruit consumption on CRC in males.

Exposure	Outcome	Nsnp	Method	β	Se	*P*-value	OR (95%CI)	Heterogeneity		Horizontal pleiotropy		
								Cochran’s Q	*P*-value	Egger intercept	Se	*P*-value
Fruit consumers in males	Colorectal cancer	7	MR Egger	0.557	1.161	0.651	1.746 (0.18-16.981)	12.377	0.054	-0.063	0.042	0.187
			Weighted median	-1.129	0.674	0.094	0.323 (0.086-1.212)					
			IVW	-0.983	0.443	0.027	0.374 (0.157-0.892)					
			Simple mode	-2.188	1.230	0.126	0.112 (0.010-1.250)					
			Weighted mode	-2.127	1.458	0.195	0.119 (0.007-2.076)					

MR, Mendelian randomization; Se, standard error; OR, odds ratio; CI, confidence intervals; IVW, inverse variance weighted.

### 3.4 MR analysis in different types of fruit

The genetic correlation between different types of fruit and CRC was investigated using a fixed effects IVW approach. Detailed information on these IVs is listed in **Supplementary Tables**.

## 4 Discussion

The causal correlation between fruit consumption and CRC was examined with a two-sample MR analysis. Our study suggests that fruit consumption may reduce the risk of CRC. This finding highlights the importance of fruit consumption in a healthy diet and the prevention of CRC.

Increasing research indicates that fruit consumption can reduce the risk of CRC. Jedrychowski et al. reported that individuals who ate one or more apples per day experienced approximately a 50% reduction in the risk of CRC (19). According to a meta-analysis of 24 studies involving 1,068,158 participants, citrus fruits, apples, watermelon, and kiwi were associated with 9%, 25%, 26%, and 13% lower risk of CRC, respectively (14). These studies illuminate the relation between fruit consumption and reduced CRC risk. Despite these correlations, our analysis across 16 types of fruit did not establish a causal correlation. The absence of a discernible causal link may be attributed to several factors. Due to the current limited extent of research on CRC, only the risk factors believed to be associated with CRC have been excluded. There may be other confounding factors, either undiscovered or lacking sufficient evidence, that have not been eliminated, thereby interfering with the relationship between fruit intake and CRC risk. Additionally, the inherently multifactorial etiology of diseases, where multiple contributors interplay, dilutes the measurable impact of individual factors. This complexity could be why the specific influence of certain fruits on CRC risk did not reach statistical significance. Therefore, to substantiate these preliminary observations, further high-quality, extensive research is essential.

The potential of fruits to reduce the risk of CRC necessitates attention to gender differences. Our study indicates that fruit consumption is protective in men, but no causal correlation was found in women. The divergent outcomes between men and women could be attributed to greater dietary heterogeneity among women, biological differences, increased measurement errors in women, different approaches to completing food frequency questionnaires between men and women, or other factors. Additionally, there are variations in health behavior characteristics between men and women within similar patterns, which might help explain why these patterns yield different outcomes (20).

Fruits are rich in various vitamins, minerals, dietary fiber, and antioxidants, such as vitamin C, vitamin A, vitamin K, potassium, magnesium, calcium, flavonoid, phenolic acid, carotenoid, and tannin, among others (21, 22). The potential biological mechanisms underlying the reduction in CRC risk associated with fruit consumption are as follows (1). Tumoricidal action: Vitamin C, polyphenol, and flavonoid exhibit toxicity towards tumor cells, inhibiting cell growth and proliferation (23, 24). Pires’ group found that pharmacological doses of vitamin C combined with certain conventional anticancer drugs can inhibit the growth of CRC cells (2, 25). Induction of apoptosis: Natural compounds such as flavonoids, terpenoids, and carotenoids have been evidenced to promote apoptosis in cancer cells (26–28). Quercetin is a flavonoid and exists in fruits like capers, apples, and berries (29). The quercetin derivative 8-C-(E-phenylmethyl) quercetin can trigger G2 phase arrest in CRC cells and inhibit proliferation. Furthermore, it induces autophagic cell death under extracellular signal-regulated kinase stimulation (3, 30). Inhibition of cell cycle progression: Flavonoids like quercetin (31) and carotenoids (32) can inhibit progression in the cancer cell cycle. Quercetin, for example, can regulate the expression of tumor suppressor genes, cycle- and apoptosis-related genes to inhibit the growth of CRC cells (4, 31). Enhanced immune system function: Components found in citrus fruits, pomegranates, and similar fruits (33), including vitamin C, polyphenolic compounds (34), and zinc (35), are believed to enhance immune system function. Additionally, researchers have found that vitamin C can increase the activity of natural killer cells (36) and T cells (37). These biological mechanisms may help explain the association between fruit consumption and a reduction of CRC risk.

A major strength of our study is that it uses large-scale GWAS to investigate causal correlations between fruit consumption and CRC. In comparison to traditional observational studies, the two-sample MR method offers a means to mitigate several common problems, including the presence of confounding factors, and the potential for biases. Then, all IVs were rigorously screened, with the lowest F-value of 20.908, indicating the accuracy of the results. Additionally, we tested for sensitivity, horizontal pleiotropy, and heterogeneity to confirm the robustness of the findings. Finally, this study utilized a CRC-related dataset, excluding patients with other types of cancer, making the conclusions more reliable.

However, the present study has some limitations. First, our findings may not apply to populations and regions beyond Europe. Studies on fruit consumption and CRC risk have varied significantly across populations and regions. A study of women in central Sweden reported an association between low fruit consumption and an increased risk of CRC (38), while a similar association was not observed in Japan (39). Second, this study did not delve into the intake frequencies and quantities. There is evidence to suggest that both low consumption and consistently low consumption of fruits may not have a significant association with CRC risk (9). Lastly, while MR analysis can reduce many confounding factors, our conclusions may be affected by environmental factors.

## 5 Conclusion

Fruit consumption may help to reduce the risk of CRC in men. Although our findings support existing public health guidelines that encourage including fruit as part of a healthy diet to lower the risk of CRC, further research is needed to validate our findings and explore the underlying mechanisms.

## Data availability statement

The original contributions presented in the study are included in the article/**Supplementary Material**. Further inquiries can be directed to the corresponding author.

## Ethics statement

Ethical approval was not required for the study involving humans in accordance with the local legislation and institutional requirements. Written informed consent to participate in this study was not required from the participants or the participants’ legal guardians/next of kin in accordance with the national legislation and the institutional requirements.

## Author contributions

LY: Conceptualization, Data curation, Investigation, Methodology, Validation, Visualization, Writing – original draft, Writing – review & editing.

## Supplementary material

Supplementary Table MR Analysis of the association between consumption of 16 different fruits and colorectal cancer risk.

## References

[1] Morgan E Arnold M Gini A Lorenzoni V Cabasag CJ Laversanne M. Global burden of colorectal cancer in 2020 and 2040: incidence and mortality estimates from GLOBOCAN. Gut. (2023) 72:338–44. doi: 10.1136/gutjnl-2022-327736 36604116

[2] Sung H Ferlay J Siegel RL Laversanne M Soerjomataram I Jemal A. Global cancer statistics 2020: GLOBOCAN estimates of incidence and mortality worldwide for 36 cancers in 185 countries. CA: Cancer J Clin. (2021) 71:209–49. doi: 10.3322/caac.21660 33538338

[3] Li X Guo D Chu L Huang Y Zhang F Li W. Potential diagnostic value of combining inflammatory cell ratios with carcinoembryonic antigen for colorectal cancer. Cancer Manage Res. (2019) 11:9631–40. doi: 10.2147/CMAR PMC6861168 32009818

[4] Wagner J Kline CL Zhou L Khazak V El-Deiry WS. Anti-tumor effects of ONC201 in combination with VEGF-inhibitors significantly impacts colorectal cancer growth and survival *in vivo* through complementary non-overlapping mechanisms. J Exp Clin Cancer research: CR. (2018) 37:11. doi: 10.1186/s13046-018-0671-0 29357916 PMC5778752

[5] Reid WV Mooney HA Capistrano D Carpenter SR Chopra K Cropper A. Nature: the many benefits of ecosystem services. Nature. (2006) 443:749. doi: 10.1038/443749a 17051186

[6] Hu Y McIntosh GH Le Leu RK Nyskohus LS Woodman RJ Young GP. Combination of selenium and green tea improves the efficacy of chemoprevention in a rat colorectal cancer model by modulating genetic and epigenetic biomarkers. PloS One. (2013) 8:e64362. doi: 10.1371/journal.pone.0064362 23717604 PMC3662759

[7] Holcombe RF Martinez M Planutis K Planutiene M. Effects of a grape-supplemented diet on proliferation and Wnt signaling in the colonic mucosa are greatest for those over age 50 and with high arginine consumption. Nutr J. (2015) 14:62. doi: 10.1186/s12937-015-0050-z 26085034 PMC4472174

[8] Michels KB Edward G Joshipura KJ Rosner BA Stampfer MJ Fuchs CS. Prospective study of fruit and vegetable consumption and incidence of colon and rectal cancers. J Natl Cancer Institute. (2000) 92:1740–52. doi: 10.1093/jnci/92.21.1740 11058617

[9] Aoyama N Kawado M Yamada H Hashimoto S Suzuki K Wakai K. Low intake of vegetables and fruits and risk of colorectal cancer: the Japan Collaborative Cohort Study. J Epidemiol. (2014) 24:353–60. doi: 10.2188/jea.JE20130195 PMC4150005 24857954

[10] Kunzmann AT Coleman HG Huang WY Cantwell MM Kitahara CM Berndt SI. Fruit and vegetable intakes and risk of colorectal cancer and incident and recurrent adenomas in the PLCO cancer screening trial. Int J Cancer. (2016) 138:1851–61. doi: 10.1002/ijc.29922 PMC6528653 26559156

[11] Lin J Zhang SM Cook NR Rexrode KM Liu S Manson JE. Dietary intakes of fruit, vegetables, and fiber, and risk of colorectal cancer in a prospective cohort of women (United States). Cancer Causes Control: CCC. (2005) 16:225–33. doi: 10.1007/s10552-004-4025-1 15947874

[12] Park Y Subar AF Kipnis V Thompson FE Mouw T Hollenbeck A. Fruit and vegetable intakes and risk of colorectal cancer in the NIH-AARP diet and health study. Am J Epidemiol. (2007) 166:170–80. doi: 10.1093/aje/kwm067 17485731

[13] Liang W Binns CW. Fruit, vegetables, and colorectal cancer risk: the European Prospective Investigation into Cancer and Nutrition. Am J Clin Nutr. (2009) 90:1112. doi: 10.3945/ajcn.2009.28320 19675109

[14] Wu ZY Chen JL Li H Su K Han YW. Different types of fruit intake and colorectal cancer risk: a meta-analysis of observational studies. World J Gastroenterol. (2023) 29:2679–700. doi: 10.3748/wjg.v29.i17.2679 PMC10198059 37213399

[15] Davey Smith G Holmes MV Davies NM Ebrahim S. Mendel’s laws, Mendelian randomization and causal inference in observational data: substantive and nomenclatural issues. Eur J Epidemiol. (2020) 35:99–111. doi: 10.1007/s10654-020-00622-7 32207040 PMC7125255

[16] Holmes MV Ala-Korpela M Smith GD. Mendelian randomization in cardiometabolic disease: challenges in evaluating causality. Nat Rev Cardiol. (2017) 14:577–90. doi: 10.1038/nrcardio.2017.78 PMC5600813 28569269

[17] Mukamal KJ Stampfer MJ Rimm EB. Genetic instrumental variable analysis: time to call mendelian randomization what it is. The example of alcohol and cardiovascular disease. Eur J Epidemiol. (2020) 35:93–7. doi: 10.1007/s10654-019-00578-3 31761964

[18] Guénard F Bouchard-Mercier A Rudkowska I Lemieux S Couture P Vohl MC. Genome-wide association study of dietary pattern scores. Nutrients. (2017) 9(7):649. doi: 10.3390/nu9070649 28644415 PMC5537769

[19] Jedrychowski W Maugeri U Popiela T Kulig J Sochacka-Tatara E Pac A. Case-control study on beneficial effect of regular consumption of apples on colorectal cancer risk in a population with relatively low intake of fruits and vegetables. Eur J Cancer Prev. (2010) 19:42–7. doi: 10.1097/CEJ.0b013e328333d0cc 19926998

[20] Reedy J Wirfält E Flood A Mitrou PN Krebs-Smith SM Kipnis V. Comparing 3 dietary pattern methods–cluster analysis, factor analysis, and index analysis–With colorectal cancer risk: The NIH-AARP Diet and Health Study. Am J Epidemiol. (2010) 171:479–87. doi: 10.1093/aje/kwp393 PMC2842201 20026579

[21] Liu RH. Health-promoting components of fruits and vegetables in the diet. Adv Nutr (Bethesda Md). (2013) 4:384s–92s. doi: 10.3945/an.112.003517 PMC3650511 23674808

[22] Rampersaud GC Valim MF. 100% citrus juice: Nutritional contribution, dietary benefits, and association with anthropometric measures. Crit Rev Food Sci Nutr. (2017) 57:129–40. doi: 10.1080/10408398.2013.862611 25831042

[23] Seeram NP Adams LS Zhang Y Lee R Sand D Scheuller HS. Blackberry, black raspberry, blueberry, cranberry, red raspberry, and strawberry extracts inhibit growth and stimulate apoptosis of human cancer cells *in vitro* . J Agric Food Chem. (2006) 54:9329–39. doi: 10.1021/jf061750g 17147415

[24] Putchala MC Ramani P Sherlin HJ Premkumar P Natesan A. Ascorbic acid and its pro-oxidant activity as a therapy for tumours of oral cavity – a systematic review. Arch Oral Biol. (2013) 58:563–74. doi: 10.1016/j.archoralbio.2013.01.016 23477602

[25] Pires AS Marques CR Encarnação JC Abrantes AM Marques IA Laranjo M. Ascorbic acid chemosensitizes colorectal cancer cells and synergistically inhibits tumor growth. Front Physiol. (2018) 9:911. doi: 10.3389/fphys.2018.00911 30083105 PMC6064950

[26] Echeverria C Santibañez JF Donoso-Tauda O Escobar CA Ramirez-Tagle R. Structural antitumoral activity relationships of synthetic chalcones. Int J Mol Sci. (2009) 10:221–31. doi: 10.3390/ijms10010221 PMC2662465 19333443

[27] Yoo KH Park JH Lee DK Fu YY Baek NI Chung IS. Pomolic acid induces apoptosis in SK-OV-3 human ovarian adenocarcinoma cells through the mitochondrial-mediated intrinsic and death receptor-induced extrinsic pathways. Oncol Lett. (2013) 5:386–90. doi: 10.3892/ol.2012.985 PMC3525461 23255955

[28] Scarano A Olivieri F Gerardi C Liso M Chiesa M Chieppa M. Selection of tomato landraces with high fruit yield and nutritional quality under elevated temperatures. J Sci Food Agricul. (2020) 100:2791–9. doi: 10.1002/jsfa.10312 PMC7187367 32022274

[29] Rauf A Imran M Khan IA Ur-Rehman M Gilani SA Mehmood Z. Anticancer potential of quercetin: A comprehensive review. Phytother Res: PTR. (2018) 32:2109–30. doi: 10.1002/ptr.6155 30039547

[30] Alzate-Yepes T Pérez-Palacio L Martínez E Osorio M. Mechanisms of action of fruit and vegetable phytochemicals in colorectal cancer prevention. Molecules (Basel Switzerland). (2023) 28(11):4322. doi: 10.3390/molecules28114322 37298797 PMC10254396

[31] Seo HS Ku JM Choi HS Choi YK Woo JK Kim M. Quercetin induces caspase-dependent extrinsic apoptosis through inhibition of signal transducer and activator of transcription 3 signaling in HER2-overexpressing BT-474 breast cancer cells. Oncol Rep. (2016) 36:31–42. doi: 10.3892/or.2016.4786 27175602 PMC4899028

[32] Ascenso A Pedrosa T Pinho S Pinho F de Oliveira JM Cabral Marques H. The effect of lycopene preexposure on UV-B-irradiated human keratinocytes. Oxid Med Cell Longev. (2016) 2016:8214631. doi: 10.1155/2016/8214631 26664697 PMC4664803

[33] Jaganathan SK Vellayappan MV Narasimhan G Supriyanto E. Role of pomegranate and citrus fruit juices in colon cancer prevention. World J Gastroenterol. (2014) 20:4618–25. doi: 10.3748/wjg.v20.i16.4618 PMC4000498 24782614

[34] González-Gallego J García-Mediavilla MV Sánchez-Campos S Tuñón MJ. Fruit polyphenols, immunity and inflammation. Br J Nutr. (2010) 104 Suppl 3:S15–27. doi: 10.1017/S0007114510003910 20955647

[35] Prasad AS. Zinc in human health: effect of zinc on immune cells. Mol Med (Cambridge Mass). (2008) 14:353–7. doi: 10.2119/2008-00033.Prasad PMC2277319 18385818

[36] Toliopoulos IK Simos YV Daskalou TA Verginadis II Evangelou AM Karkabounas SC. Inhibition of platelet aggregation and immunomodulation of NK lymphocytes by administration of ascorbic acid. Indian J Exp Biol. (2011) 49:904–8. 22403863

[37] Grant MM Mistry N Lunec J Griffiths HR. Dose-dependent modulation of the T cell proteome by ascorbic acid. Br J Nutr. (2007) 97:19–26. doi: 10.1017/S0007114507197592 17217556

[38] Terry P Giovannucci E Michels KB Bergkvist L Hansen H Holmberg L. Fruit, vegetables, dietary fiber, and risk of colorectal cancer. J Natl Cancer Institute. (2001) 93:525–33. doi: 10.1093/jnci/93.7.525 11287446

[39] Sato Y Tsubono Y Nakaya N Ogawa K Kurashima K Kuriyama S. Fruit and vegetable consumption and risk of colorectal cancer in Japan: The Miyagi Cohort Study. Public Health Nutr. (2005) 8:309–14. doi: 10.1079/PHN2004681 15918928

